# 3D printing and bioprinting in the battle against diabetes and its chronic complications

**DOI:** 10.3389/fbioe.2024.1363483

**Published:** 2024-05-28

**Authors:** Indumathi Sathisaran

**Affiliations:** Department of Bioscience and Engineering, National Institute of Technology Calicut, Kozhikode, Kerala, India

**Keywords:** diabetes, 3D printing, 3D bioprinting, chronic complications, diagnosis, treatment, cell-seeded hydrogels, diabetic disease models

## Abstract

Diabetes is a metabolic disorder characterized by high blood sugar. Uncontrolled blood glucose affects the circulatory system in an organism by intervening blood circulation. The high blood glucose can lead to macrovascular (large blood vessels) and microvascular (small blood vessels) complications. Due to this, the vital organs (notably brain, eyes, feet, heart, kidneys, lungs and nerves) get worsen in diabetic patients if not treated at the earliest. Therefore, acquiring treatment at an appropriate time is very important for managing diabetes and other complications that are caused due to diabetes. The root cause for the occurrence of various health complications in diabetic patients is the uncontrolled blood glucose levels. This review presents a consolidated account of the applications of various types of three-dimensional (3D) printing and bioprinting technologies in treating diabetes as well as the complications caused due to impaired blood glucose levels. Herein, the development of biosensors (for the diagnosis), oral drug formulations, transdermal drug carriers, orthotic insoles and scaffolds (for the treatment) are discussed. Next to this, the fabrication of 3D bioprinted organs and cell-seeded hydrogels (pancreas engineering for producing insulin and bone engineering for managing bone defects) are explained. As the final application, 3D bioprinting of diabetic disease models for high-throughput screening of ant-diabetic drugs are discussed. Lastly, the challenges and future perspective associated with the use of 3D printing and bioprinting technologies against diabetes and its related chronic complications have been put forward.

## Graphical Abstract

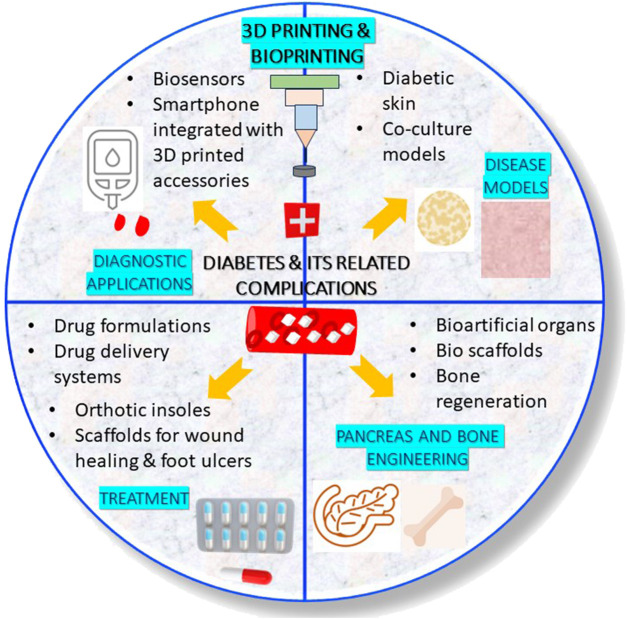

## 1 Introduction

Diabetes, commonly referred to as a “silent-killer disease” or a “rich man’s disease” has been growing as a major chronic disease of concern in recent times (Khan et al., 2019; India Today, 2023). According to International Diabetes Federation (IDF), 10.5% of population at the age group of 20–79 years has diabetes and half of the population among this 10.5% is unaware about the possession of the disease (International Diabetes Federation, 2024). The study by IDF also reported that by the year 2045, it is expected that one in eight adults (equivalent to 783 million) will be diabetic (International Diabetes Federation, 2024). Diabetes is a metabolic disorder characterized by higher concentrations of blood sugar/glucose levels. Pre-diabetes is a kind of metabolically disordered health condition, where the individual possesses high blood sugar level than the normal range (Khan et al., 2019). Although the pre-diabetic condition is characterized by high blood glucose, the detected high blood sugar level might not be appropriate enough to confirm the diabetic condition. Diabetes has been classified into three types, namely, the type 1 diabetes (T1D) where the body is unable to produce enough insulin), type 2 diabetes (T2D) or non-insulin dependent diabetes (where the body produces insulin but is unable to use it effectively) and gestational diabetes (diabetes developed during pregnancy) (Centers for Disease Control and Prevention, 2023).

The high blood glucose initially affects the large blood vessels (leading to macrovascular complications), and if left undiagnosed, also starts affecting the small blood vessels (resulting in microvascular complications). As high blood sugar can bring down the immunity in an individual, it has to be noted that a diabetic patient is susceptible for acquiring infections often than a healthy person (Berbudi et al., 2020). Hence, the treatment measures need to be taken from the beginning stage of diagnosis of diabetes or pre-diabetes. Acquiring treatment at an appropriate time in the early stage is very important for the effective management of diabetes.

In recent years, 3D printing and 3D bioprinting are gaining attention as forefront technologies in diagnosis and treatment of various disorders/diseases. These technologies are extensively used for the production of pharmaceutical drugs, medical products and artificial organs (Cui et al., 2021; Yi et al., 2021; Tripathi et al., 2023). Chakka and Chede (2023) define the term “3D” in “3D Printing and Bioprinting” as “Design,” “Develop” and “Dispense” (Chakka and Chede, 2023). The technology comprises the creation of a three-dimensional (3D) object from a CAD/digital 3D model. 3D printing and 3D bioprinting technologies, despite holding the similar working principle, differ from each other in terms of the composition of the ink used for printing the objects. 3D printing is used for printing solid materials and hence the composition of printing ink is polymers and chemicals. On the other hand, 3D bioprinting is used for the production of substrates, artificial organs and *in vivo* models that mimics the cellular microenvironment in living organisms. The ink used in 3D bioprinting process consists of biological materials such as cells/living tissues along with (bio) polymers, growth media, regulators, etc. And hence the ink used in a 3D bioprinting process is referred to as “bioink.”

3D printing and bioprinting technologies are used in various ways in the diagnosis and treatment of diabetes. This review highlights the recent applications of 3D printing and bioprinting in treatment of diabetes and some of the common health complications caused due to diabetes. Role of 3D printing and 3D bioprinting technologies in the diagnosis and treatment of diabetes (hyperglycemia) and its associated chronic complications (especially neuropathy, hypertension, dyslipidemia, peripheral diabetic neuropathy, diabetic wounds, diabetic foot ulcers), engineering of pancreas (to overcome graft rejection due to pancreas transplant) and bones (to treat and manage bone fracture and inhibition of osteogenesis) and fabrication of 3D drug testing models for rapid screening of anti-diabetic drugs have been discussed. Lastly, a glimpse of challenges and future perspective associated with 3D printing and 3D bioprinting in the management and treatment of diabetes is discussed.

## 2 Types of 3D printing

**FIGURE 1:**
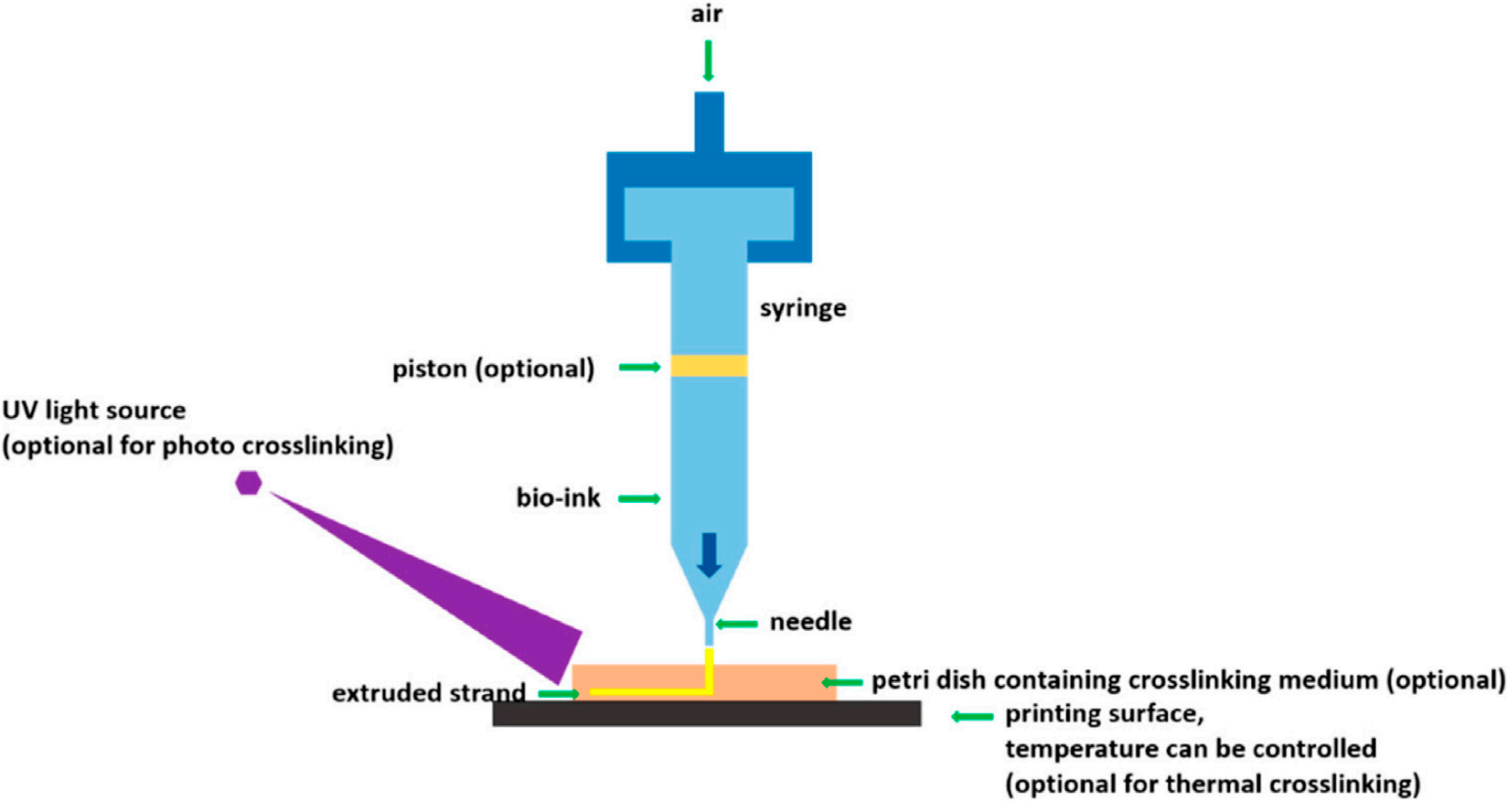
Schematic of an extrusion-based 3D bioprinter [Reproduced from (You et al., 2017] with permission. Copyright 2017 MDPI).

**FIGURE 2:**
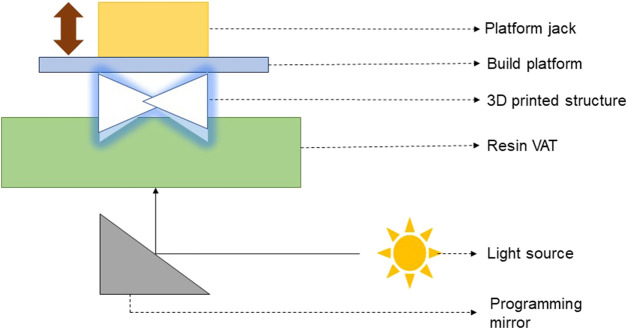
Schematic of the working principle of a SLA 3D printer/bioprinter (Verisqa et al., 2022).

**FIGURE 3:**
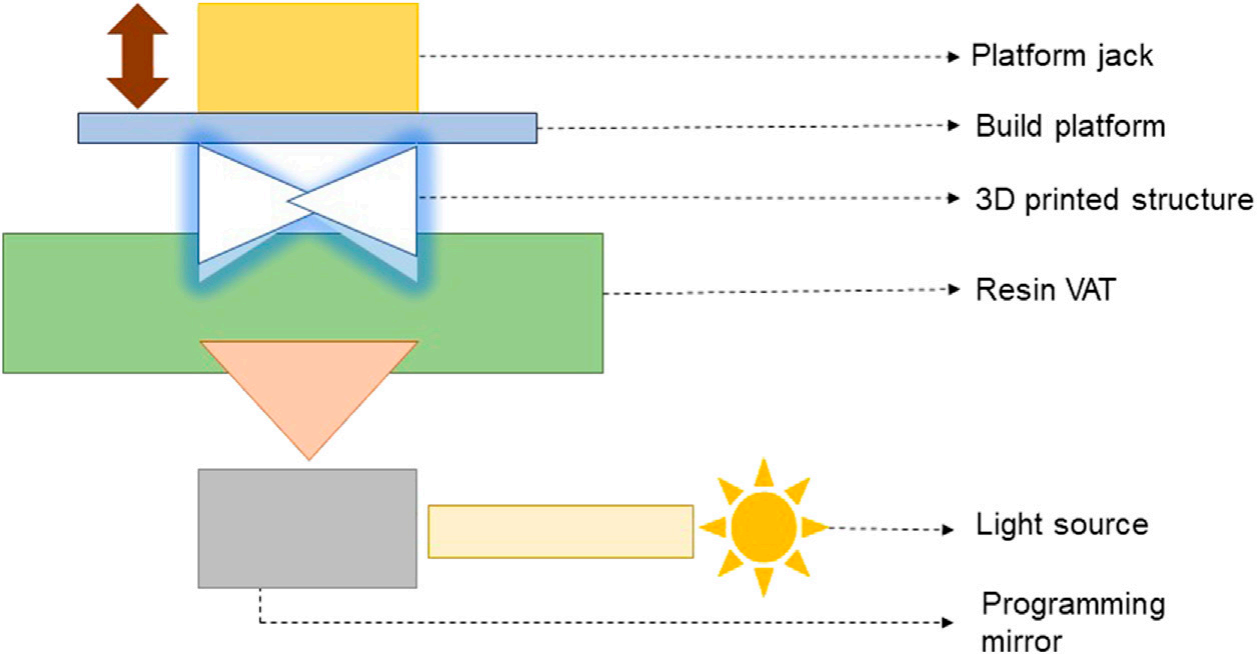
Schematic of the working principle of a DLP-based 3D printer/bioprinter (Verisqa et al., 2022).

Different types of 3D printing strategies have been used in the production of pharmaceuticals or biomedical devices for the diagnosis and treatment of diabetes. Extrusion-based 3D printing, Fused Deposition Modeling (FDM), Co-axial extrusion-based 3D printing, Stereolithography (SLA) 3D printing, Digital Light Processing (DLP)-based 3D printing, Selective Laser Sintering (SLS) and Ink-Jet Printing (IJP) are the various kinds of 3D printing and bioprinting techniques used in the development of pharmaceuticals and biomedical products. The working principle of some of the 3D printing approaches are described below:

*(a)* *Extrusion-based 3D printing and bioprinting*: Extrusion-based bioprinting has been evolving as one of the leading approaches for the manufacture of pharmaceuticals, regenerative medicine and tissue engineering. The principle behind the working of an extrusion-based 3D bioprinter is that ink [drug(s) loaded in the polymeric gel] or bioink (polymer with biological materials) will be extruded through the nozzles to create 3D structures. The so formed 3D bioprinted structures can be further crosslinked for obtaining final 3D structures. Figure 1 presents the schematic of the working principle of an extrusion-based 3D bioprinter.
*(b)* *Fused Deposition Modeling (FDM)*: FDM, also referred to as “Fused Filament Fabrication” is a process in which the solid/thermoplastic filaments are extruded in a layer-by-layer manner through a nozzle after melting to form a 3D-object (Acierno and Patti, 2023). The main requisite of the printing material for its use in FDM 3D printing is that the molten printing material should be capable of solidifying immediately once the printing is over. FDM is mostly used in the continuous production of pharmaceuticals.
*(c)* *Co-axial 3D bioprinting*: Co-axial 3D bioprinting is a strategy that facilitates multimaterial printing that simultaneously dispenses multiple bioinks through a single filament (containing concentric orifices to dispense bioinks concentrically). This kind of 3D printing strategy is mostly used in biofabrication of cell-laden constructs/tissue engineering applications (Hong et al., 2019; Kim and Nam, 2020; Liu et al., 2022) and to a certain extent in the manufacture of pharmaceutical formulations (Talebian et al., 2021). An interested reader can explore for more details on the applications of co-axial 3D bioprinting in tissue engineering by referring to the review by (Kjar et al., 2021).
*(d)* *SLA 3D printing and bioprinting*: SLA, also known as “vat polymerization” 3D printing is a process in which the photosensitive resin in the form of liquid is poured into a tank and cured by UV light for solidification (Dassault Systèmes, 2024). SLA 3D printing and bioprinting techniques are mostly used in the fabrication of pharmaceutical formulations and cell scaffolds, especially the bone scaffolds. Figure 2 presents the schematic of principle behind the working of a SLA 3D printer/bioprinter.
*(e)* *DLP-based 3D printing and bioprinting*: In DLP-based 3D bioprinting, the digital light source is allowed to project layer-by-layer in the form of surface light on a liquid photosensitive resin surface, followed by solidification of layers. Figure 3 presents the schematic of the working principle of a DLP-based 3D printer/bioprinter.

## 3 Applications of 3D printing and 3D bioprinting in diabetic patients

### 3.1 Diagnostic applications

#### 3.1.1 Biosensors and other analytical tools

Biosensors are analytical devices used in sensing and detection of high glucose level in diabetic patients. Liu et al. (2021) devised and investigated the utility of MN-based biosensor for subcutaneous-level interstitial glucose sensing applications (see Section 3.2.2.) (Liu et al., 2021). Remarkably, Goel and co-workers developed a portable platform using a combination of 3D printing, laser (CO_2_ laser) and electrode (graphene electrode) technologies that could detect the sugar level in patients with type I and type II diabetes (Kumar et al., 2023). Very recently, Piaggio et al. (2024) proposed a smart-tool for screening diabetic neuropathies based on the integration of 3D printed accessories and a smartphone app. The findings from the experiments conducted with 11 normosubjects established that the smart-tool could serve as a practical solution to improve the SoC of diabetic patients suffering with diabetic neuropathy (Piaggio et al., 2024).

### 3.2 Therapeutic applications

#### 3.2.1 Oral medications

3D printing has been recognized as one of the promising strategies implemented for the production of oral solid dosage forms (Ventola, 2014; Ahmad et al., 2023). Various printing methods such as 3D extrusion-based printing (Ahmed et al., 2021), Fused Deposition Modeling (FDM) (Ibrahim et al., 2019), Ink-Jet Printing (IJP) (Stranzinger et al., 2021) and Semi-Solid Extrusion (SSE) printing (Cui et al., 2019) have been used by researchers for the production of various types of oral formulations. Khaled et al. (2015) employed 3D extrusion-based printing and produced polypills containing captopril, nifedipine and glipizide with controlled release profiles. These polypills potentially find its use in the treatment of hypertension in diabetic patients (Khaled et al., 2015).

Ahmed et al. (2021) reported Self-Nano Emulsifying Drug Delivery System (SNEDDS)-based 3D-printed tablet formulation containing GLMP and rosuvastatin (RSV) for treating dyslipidemia in diabetic patients (Ahmed et al., 2021). Extrusion-based 3D printing method was employed for the preparation of the formulation. The comparison of SNEDDS tablets with non-SNEDDS tablets indicated that the drug release behaviour of SNEDDS tablets were superior to that of the non-SNEDDS tablets (Ahmed et al., 2021). The researchers surmised that the formulated SNEDDS-based tablets could serve as a promising combined oral medication for diabetes treatment.

Ibrahim et al. (2019) 3D printed Metformin HCL tablets using Poly Vinyl Alcohol (PVA) as polymer by FDM and investigated the drug loading, tablet design and dissolution studies for those 3D printed tablets (Ibrahim et al., 2019). Likewise, Gioumouxouzis et al. (2018) 3D printed bilayer oral solid dosage form combining metformin for prolonged and glimepiride (GLMP) by FDM for immediate drug delivery (Gioumouxouzis et al., 2018). Stranzinger et al. (2021) used Near-Infrared (NIR) hyperspectral imaging as a monitoring tool for on-demand manufacturing of ink-jet printed metformin hydrochloride formulations (Stranzinger et al., 2021).

Cui et al. (2019) utilized SSE 3D printing and fabricated tablets with customized internal structures containing personalized doses in it (Cui et al., 2019). The researchers used glipizide as model drug and investigated the effect of three different concentrations of the polymer HPMC K100LV polymer in the 3D printing of glipizide tablets (Cui et al., 2019). The study revealed that the tailored drug release was achieved with the 3D printed tablets, thereby proving that personalized tablets prepared by SSE 3D printing can be effectively used to modulate the physicochemical properties of drug (Cui et al., 2019).

#### 3.2.2 Transdermal drug delivery systems

Transdermal drug delivery has been gaining attraction as an effective drug delivery system since it offers several advantages such as being patient friendly, non-invasive, and potential to bypass the first-pass metabolism by liver (Alkilani et al., 2015; Wong et al., 2023). It is not astonishing to see that the application of transdermal drug delivery system has also been progressing in the battle against diabetes.

Pere et al. (2018) reported the fabrication of polymeric microneedle (MN) patches containing insulin by means of (SLA) printing (Pere et al., 2018). A biocompatible class I resin, Dental SG was photopolymerized into pyramid and cone designs. Insulin was loaded into three carbohydrate-based drug carriers, namely, the trehalose, mannitol and xylitol, which were further inkjet print-coated onto the pyramid and cone MN designs. Effect of MN design on the insulin release and the integrity of the drug carrier containing insulin were evaluated. The three carriers were reported to maintain good integrity with xylitol exhibiting best performance and insulin release was found to be rapid with both the pyramid and cone designs. The researchers suggest that the proposed biocompatible 3D printed-MNs would be biocompatible as well as scalable (Pere et al., 2018).

Analogous to the discussed work above, Economidou et al. (2019) fabricated 3D printed MN patches with superior skin penetration capacity by photopolymerizing biocompatible resin layer-by-layer in pyramid and spear designs, for intradermal delivery of insulin by means of stereolithography (SLA) (Economidou et al., 2019). Carrier made up of sugar, and insulin were coated onto the surface of resin by Ink-Jet Printing. The researchers concluded that the fabricated MN system was able to exhibit fast insulin action and well-controlled hypoglycemic condition during the *in vivo* trial experiments with diabetic mice. Following this study, the same research team worked on the optimization of design and manufacturing parameters of 3D printed solid MNs of insulin for improved strength, sharpness, and delivery (Economidou et al., 2021). It was illustrated that the coating morphology, piercing behaviour and fracture of the MNs were influenced by its geometry (Economidou et al., 2021).

Interestingly, very limited number of reports have explored the formulation of MN system by extrusion-based 3D printing. Wu et al. (2020) demonstrated the employability of extrusion-based 3D printing in the fabrication of insulin-loaded MN patch for regulated delivery of insulin and glucose management in diabetic mice (Wu et al., 2020).

A step ahead of transdermal drug delivery, Liu et al. (2021) explored the use of MNs subcutaneous-level interstitial glucose sensing applications (Liu et al., 2021). A biosensor constructed by the integration of four techniques, namely, 3D printing, microfabrication, an electroplating and an enzyme immobilization was investigated for the sensing, continuous and painless monitoring of interstitial glucose level in mice (Liu et al., 2021). The MNs-based biosensing device exhibited a consistent and steady detection of glucose in various biological fluids such as in buffer, plasma, and simulated interstitial fluid (ISF) (Liu et al., 2021). The authors proposed that the electroplating and microfabrication techniques aided the sensor to sense and detect glucose with good sensitivity in a broad detection range (Liu et al., 2021). This finding could possibly pave for the invention of new analytical devices for continuous monitoring of blood glucose levels which will be extremely useful one for diabetic patients.

#### 3.2.3 Orthotic insoles

50% of diabetic patients were believed to develop symptomatic peripheral neuropathy within 25 years of first occurrence of diabetes (Volmer-Thole and Lobmann, 2016). Peripheral diabetic neuropathy is one of the major chronic complications that affects feet, legs, digestive system and heart (Zaino et al., 2023). However, the organ system that often gets affected because of this neuropathy is the nervous system of the legs and feet. The orthotic insoles are special kind of biomedical product that finds its application in the treatment of foot-related complications caused because of peripheral diabetic neuropathy among diabetic patients. Foot insoles are used in relieving pains (especially site-specific pains) in persons hailing diabetic foot complications. Plantar pressures (or distribution of loads on foot) in diabetic patients act as one of the important parameters in evaluating the progression of diabetes (Zuniga et al., 2022). Custom insoles, also referred to as “Standard of Care (SoC) insoles” are well-known for its use in dealing with diabetic foot plantar pressures. Though these SoC insoles are customized, these still do possess a drawback of being dependent on the generic properties of materials. However, recent research has evidenced the 3D Printing strategy could be employed to circumvent this drawback by relying on individualized materials in the fabrication of diabetic-friendly personalized insoles (Mancuso et al., 2023). Mancuso et al. (2023) conducted a pilot study named “DIAPASON” (DIAbetic PAtients Safe ambulatiON) and studied the safety of 3D-printed insoles, when used by an italian patient with foot complications associated with T2D in an ambulatory setting (Mancuso et al., 2023).

In the past few days, integration of 3D printing, scanning, materials chemistry and software application have been highly beneficial in fine-tuning the procedure of insole fabrication process and in enhancing the performance of insoles as well (Zuniga et al., 2022; Mancuso et al., 2023). For instance, Zuniga et al. (2022) developed 3D-printed insoles employing the thermoplastic polyether-polyurethane and thermoplastic polyurethane polyester-based polymer for diabetic patients and evaluated its efficiency in plantar pressure distribution with electronic pressure sensors (Zuniga et al., 2022). The insoles were found to perform well and were reported to possess potential in managing diabetes. The patients also provided feedback that the insoles made up of thermoplastic polyurethane polyester-based were comfortable than that of the standard insole and the insole made up of the thermoplastic polyether-polyurethane. Similarly, Muir et al. (2022) evaluated the plantar distribution potential of 3D printed accommodative insoles (Muir et al., 2022). Patient-specific metamaterials were used in the 3D printed insole design. Likewise, Hudak et al. (2022) developed a novel workflow to fabricate patient-specific 3D printed accommodative foot orthosis with personalized lattice metamaterial (Hudak et al., 2022).

Maharana et al. (2022) proposed a kind of 3D printed, mechanically operated dynamically self-offloading customized therapeutic footwear that involves no use of sensors and actuators (Maharana et al., 2022). The foot dimensions and walking style were customized in the therapeutic footwear made of the polymer, thermoplastic polyurethane (Maharana et al., 2022). The working principle of the therapeutic footwear was based on the mechanism that when a load is applied, the snapping arches in the footwear enters the negative-stiffness regime. In contrast, offloading the load will snap to a different shape, followed by reverting back of the snapping arch to the initial shape when the load disappears (Maharana et al., 2022). The research team hypothesize that the snapping mechanism helps to keep the feet well-balanced, and also assist in quicker healing of the injured sites of the feet (Inidian institute of science, 2022; Maharana et al., 2022).

#### 3.2.4 Diabetic wounds and foot ulcers

Diabetic wounds are another kind of complications that affects the quality of life of most of the diabetic patients with peripheral neuropathy. A typical wound healing process comprises four important phases, namely, hemostasis, inflammation, proliferation and remodeling (Guo and DiPietro, 2010; Huang et al., 2022). Unlike in non-diabetics, the blood circulation gets slowdown in diabetic patients and hence the body’s efficiency to deliver nutrients to the wound regions gets disrupted. This causes the diabetic wounds to heal slowly. Moreover, the diabetic wounds are also associated with high infection risk. Similar to wounds, some diabetic patients also suffer with slow healing foot ulcers, commonly referred to as “Diabetic Foot Ulcers (DFU).” While diabetic wounds are caused by cuts or abrasions, DFU starts as wounds and further progresses as foot sore with poor ability to heal. DFU is a kind of skin lesion caused because of the neuropathic complications in T1D and T2D patients when sugar level is not under control. DFU, if left untreated may lead to amputation (Kushmakov et al., 2018). The impairment of wound healing or DFU in diabetes is mainly attributed to poor immune response (NIH Research Matters, 2020), poor glycemic control (Game, 2008), renal failure (Game, 2008) and visual impairment (Game, 2008). Hence, early detection and treatment for DFU is very much important. For past few years, 3D bioprinting has been explored by several researchers in fabricating scaffolds for wound healing. Our literature survey indicate that good number of review articles have been contributed by researchers in this regard (Tsegay et al., 2022; Freedman et al., 2023; Pita-Vilar et al., 2023; Uchida and Bruschi, 2023). This section presents the recent applications of 3D bioprinting in the production of scaffolds for diabetic wound healing.

**FIGURE 4:**
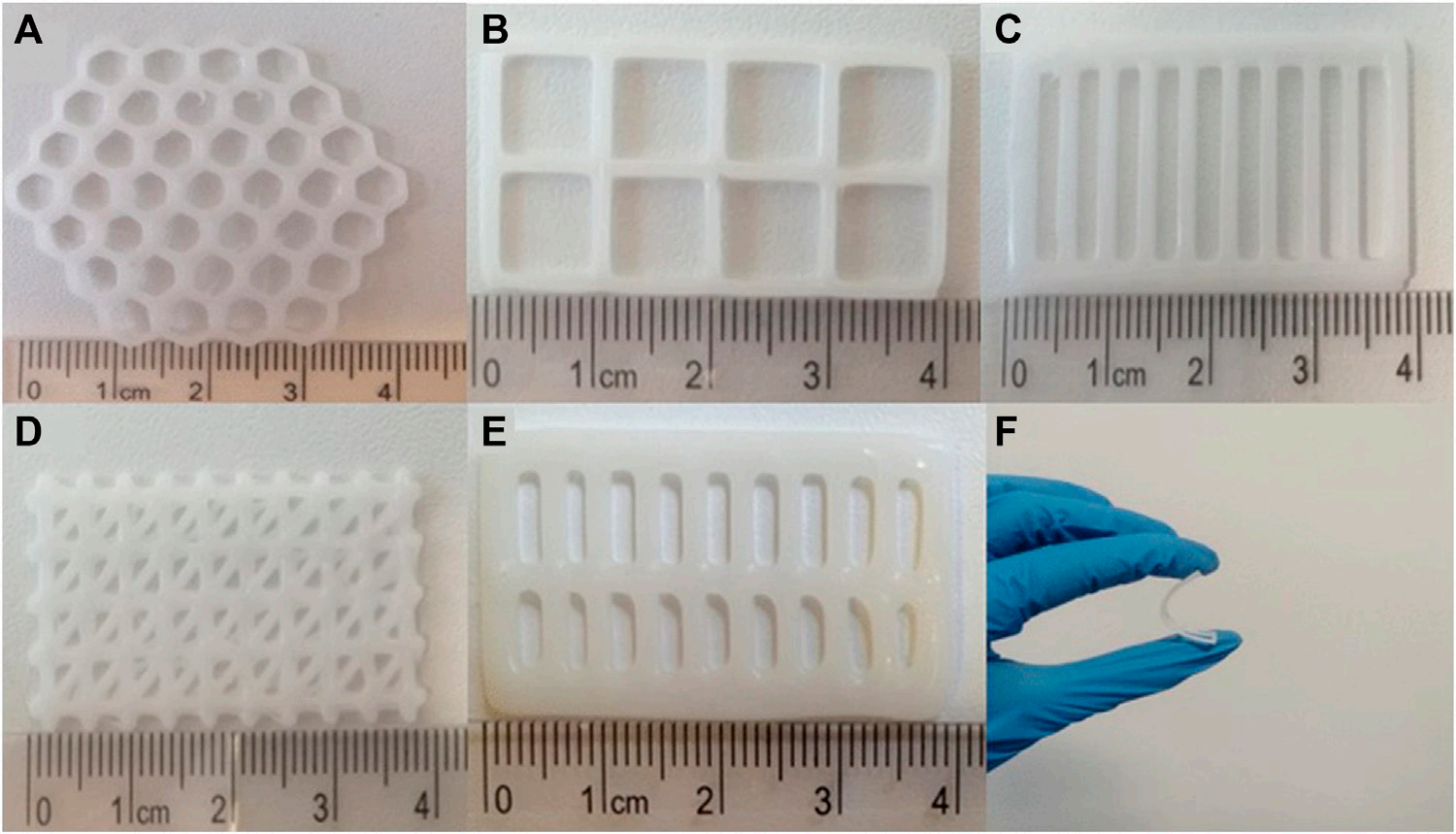
Digital images of 3D bioprinted PCL scaffolds of different designs: **(A)** honeycomb, **(B)** square, **(C)** parallel, **(D)** triangular, **(E)** double-parallel, and **(F)** the fexibility a bioprinted scaffold [Reproduced from (Glover et al., 2023) with permission. Copyright 2023 Springer Nature].

Fratini et al. (2023) integrated microfluidics technology with co-axial 3D bioprinting to fabricate a wound dressing that can simultaneously reduce the bacterial load and promoting wound healing (Fratini et al., 2023). Customization of a wound-healing scaffold based on the nature of wound in a patient can help in managing patient’s demand efficiently. Accordingly, Glover et al. (2023) fabricated 3D bioprinted polycaprolactone (PCL) scaffolds of different designs (honeycomb, square, parallel, triangular and double-parallel) loaded with an antibiotic, levoflocixin and investigated the sustained drug release for wound-healing applications (Glover et al., 2023). The impact of scaffold geometry and antibiotic concentration on the mechanical properties were examined. Figure 4 presents the digital images of 3D bioprinted PCL scaffolds of various designs and the fexibility a bioprinted scaffold. Also, the drug release characteristics of scaffolds were investigated in the study. The researchers established that the scaffolds can be modified according to the size of the wound and can serve as a potential strategy for treating DFU when compared with that of the conventional treatment strategies as it could perfectly meet the patient’s demand by being simpler and economical (Glover et al., 2023). Jiang et al. (2023), recently contributed an informative review on the present status and progress on dressing management for DFU (Jiang et al., 2023). Table 1 presents the summary of literature reports available on the 3D bioprinting of scaffolds fabricated for healing of diabetic wounds and DFU.

**TABLE 1:** Summary of literature reports available on 3D bioprinted hydrogels/scaffolds fabricated for diabetic wound healing and DFU healing applications.

S. No.	Type of the 3D printed biomaterial	Hydrogel composition	Fabrication method	Comments	Ref(s)
01	Scaffold	Inner core of the scaffold: nanocomposite hydrogel composed of hydroxyethyl cellulose (HEC) and PEGylated LPs encapsulated with thyme oil (TO) prepared by microfluidics technology with an Active Pharmaceutical Ingredient (API); Outer core—a hybrid hydrogel composed of sodium alginate/cellulose nanocrystals (SA/CNC) and enriched with free TO	Integration of microfluidics technology with co-axial 3D-bioprinting	The scaffolds exhibited a combination of burst and sustained release of drug; The incorporation of bioactive compound in the scaffold imparted the anti-bacterial properties to the scaffold	Fratini et al. (2023)
02	Antibiotic scaffold	Levoflocixin	Extrusion-based bioprinting using a Bio-X bioprinter thermoplastic print head	The antibiotic scaffolds showed excellent mechanical properties and exhibited a sustained drug release for 4 weeks	Glover et al. (2023)
03	Porous scaffolds	Chitosan	Extrusion-based 3D printing process	Improvement in the quality of the restored tissue during the healing process in streptozotocin-induced diabetic rats	Intini et al. (2018)
04	MoS_2_ accelerated gelling hydrogel scaffold	MoS_2_ nanosheets, benzaldehyde and cyanoacetate group-functionalized dextran solution	*in situ* three-dimensional (3D) bioprinting	Assistance in the closure of wounds, eased the oxidative stress, eliminated bacterial infection and positively improved the wound healing process	Ding et al. (2023)
05	Wound dressing	DNA from salmon sperm and DNA-induced biosilica	Artificial Intelligence (AI)-based 3D bioprinting	Enhancement of the biological activity of the dressings through scavenging of reactive oxygen species (ROS); Promotion of angiogenesis; Anti-inflammation property; Acceleration of the acute and diabetic wound healing	Kim et al. (2023)
06	3D-bioprinted autologous adipose tissue grafts	Fibrin glue	3D bioprinter	Promotion of wound healing with high-quality reconstruction of skin tissues	Bajuri et al. (2023)
07	Scaffolds	Satureja cuneifolia plant extract (SC), sodium alginate (SA)/polyethylene glycol (PEG)	3D printing	Best antibacterial activity (mainly against gram-positive bacteria); Promotion of diabetic wound healing	Ilhan et al. (2020)
08	Injectable amyloid-based composite hydrogel and 3D printable hydrogel	Bovine serum albumin and aloe vera	3D printing	Provision of best shape fidelity and mechanical properties suitable for faster chronic wound healing	Naik et al. (2023)
09	Multicomponent biocomposite hydrogel wound dressings	Chitosan methacrylamide, cellulose nanocrystal, antibacterial silver nanoparticles and vascular endothelial growth factor	3D Printing	Improvement in granulation tissue formation and differential points of vascular density; Yielding of various physiological responses in mouse model depending on the growth factor	Alizadehgiashi et al. (2021)
10	MeHA patches	Methacrylated hyaluronic acid (MeHA) and small extracellular vesicles (sEVs) attained from human mesenchymal stem cells (MSC-sEVs)	Extrusion-based 3D printing process	Improvement in wound closure in diabetic mouse	Ferroni et al. (2023)
11	Scaffolds	Copper-epigallocatechin gallate (Cu-EGCG) capsules loaded in a methacrylated decellularized extracellular matrix-based hydrogel	Extrusion-based 3D printing process	The dermal scaffolds exhibited a good pore size, excellent biocompatibility and promoted angiogenesis	Hu et al. (2023)
12	Hydrogel	Bioactive elements, egg white	3D Printing	The hydrogels stimulated the fibroblasts and adipose tissue-derived stem cells without imparting any cytotoxic effects	Guo et al. (2022)
13	Core-shell hydrogel microfiber	Poly Vinyl Alcohol/Indomethacin/MMP inhibitor (PVA/INDO/MMPI) functional bio-ink	Co-axial biological 3D printing	The microfiber-based dressings possessed multifunctional properties such as controlled drug-release, excellent water absorption (and water retention), good biocompatibility, wound-healing, antibacterial and anti-inflammatory properties	Huang et al. (2022)
14	Peptide-based hydrogel	Thiolated γ-polyglutamic acid (γ-PGA-SH), glycidyl methacrylate-conjugated γ-polyglutamic acid (γ-PGA-GMA), thiolated arginine-glycine-aspartate (RGDC) sequences and vascular endothelial growth factor 165-overexpressed human umbilical vein endothelial cells	3D bioprinting	The cell-laden hydrogel promoted angiogenesis, reduced tissue hypoxia and minimized inflammation	Huang et al. (2023)
16	Composite scaffolds	Gelatin-decellularized matrix—quaternized chitosan [Gel-dECM-Qcs (GDQ)] bioink	Extrusion-based 3D printing process	GDQ composite scaffolds exhibited good mechanical properties, good biocompatibility, wound healing, and antimicrobial ability	Zhong et al. (2023)
17	Scaffold	Decellularized small intestinal submucosa (SIS) combined with mesoporous bioactive glass (MBG) and exosomes	3D Printing in low temperature	Acceleration of diabetic wound healing and induction of angiogenesis by the scaffolds; Promotion of granulation tissue formation, collagen fiber deposition, and growth of functional new blood vessels by the scaffolds	Hu et al. (2021)

### 3.3 Engineering of pancreas and bones

#### 3.3.1 3D bioprinted organs, bio scaffolding/encapsulation systems

**FIGURE 5:**
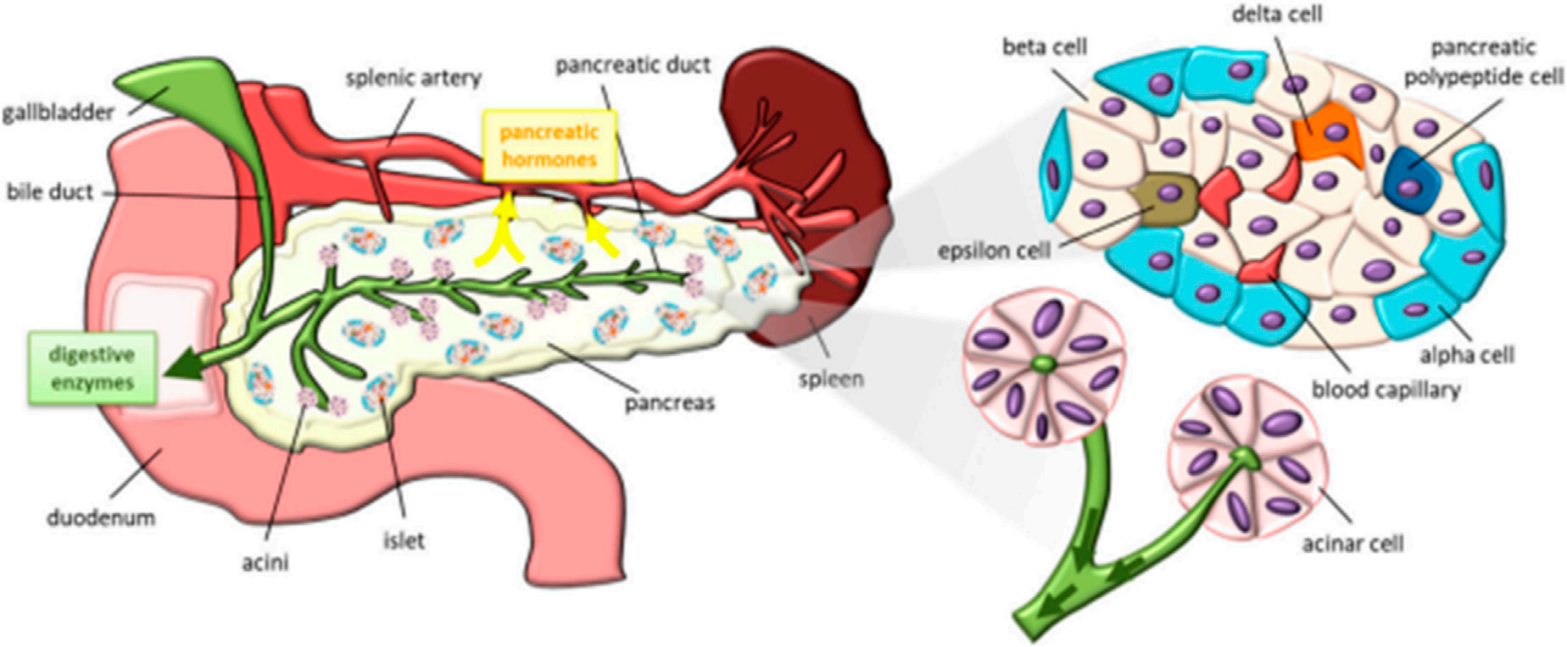
Schematic representation of a pancreas and its constituents [Reproduced from (Moulle and Parnet, 2019) with permission. Copyright 2019 MDPI].

Among the various organs in the organ system, pancreas play a vital role in maintaining blood glucose level (Sharabi et al., 2015; Roder et al., 2016) by secreting the glucose-maintaining hormones, insulin and glucagon. A pancreas is an elongated organ localized behind the stomach and across the belly. A pancreas consists of an exocrine chamber of secretory cells that synthesizes digestive juice, further releases the juice into pancreatic duct, and an endocrine chamber of islet cells. These islet cells include α cells and β cells that do produce two vital hormones, glucagon and insulin for maintaining and regulating the blood glucose level (Moulle and Parnet, 2019). A schematic demonstrating the structure and constituents of a pancreas is shown in Figure 5.

When the function of a pancreas or liver is impaired, the regular glucose metabolism in the body will be disrupted, thereby leading to pre-diabetes or diabetes. Though chemotherapy via intake of drugs such as metformin or insulin therapy can be adopted as a treatment for T2D (the condition where the body is unable to use the produced insulin) or T1D (the body is unable to produce enough insulin) the drug-based or insulin therapy may become inadequate for patients during the course of time. In such a condition, the patients will be in need of pancreatic islets or pancreas transplant (Wszola et al., 2021). Islet transplantation is in forefront among the various strategies available for treating T1D. However, islet transplantation may unfortunately fail due to inevitable reasons such as immune response directed by the host to the transplanted cells, low viability and low functionality of islet cells.

**FIGURE 6:**
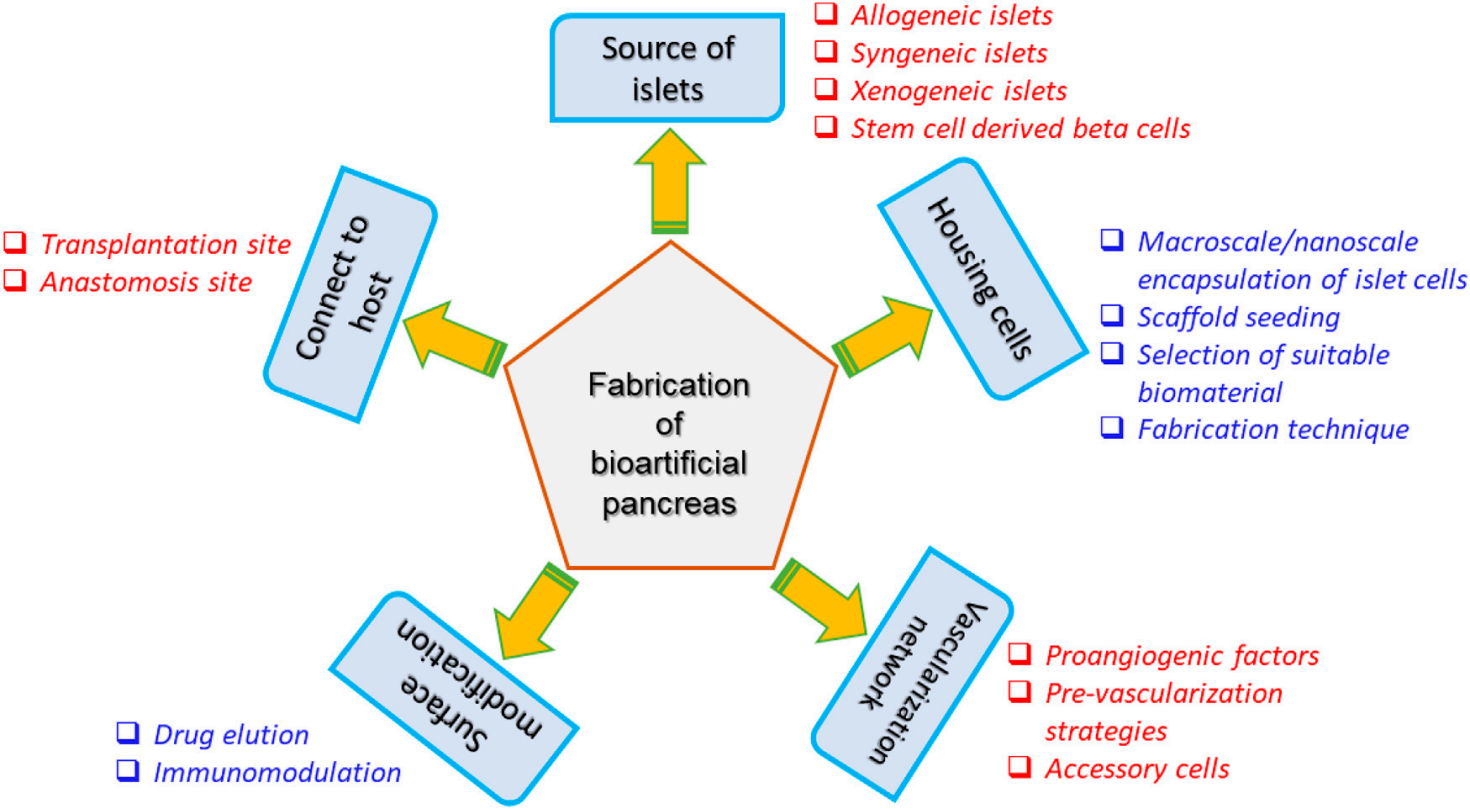
Considerations for the fabrication of bioartificial pancreas (Soetedjo et al., 2021).

Transplantation of bioartificial organs (bioartificial pancreas) is an attractive strategy that can be employed to circumvent the above-mentioned drawbacks. Fabrication of a bioartificial pancreas is a complicated process and utmost care should be taken with respect to several aspects notably source of islet cells, biocompatibility to the host, possession of vascularization network and enriched nutrient supply. Figure 6 presents the schematic indicating the various factors that influence the fabrication of a bioartificial pancreas (Soetedjo et al., 2021).

3D Bioprinting can be employed to get rid of the drawback of graft rejection by the body through means of facilitating donor-independent T1D treatment strategy. A polymer chosen for bioprinting organs should possess the vital characteristics such biodegradable, biocompatible, 3D printable, crosslinkable and storable. In donor-independent T1D treatment strategy of restoring glucose homeostasis, the human stem cells will be cultured *in vitro* and differentiated into β cells (insulin-producing cells) and α cells (glucagon-producing cells). The β and α cells will be impregnated into the bioink for the 3D bioprinting of pancreas, which will be transplanted into patients (Wszola et al., 2021). Accordingly, Farina et al. (2017) fabricated a functionalized encapsulation system and examined its utility in the subcutaneous engraftment of islet-like cells (Farina et al., 2017). The encapsulation system was to offer protection against acute hypoxia, enhanced hydrophilicity, good cell attachment and proliferation properties (Farina et al., 2017).

The components in Extracellular matrix (ECM) influence the biological activity, mechanical stability and life span of the *in vitro* pancreatic culture (Daoud et al., 2011). This demands the fine-tuning of composition of ECM to ensure long-term usage and preservation of *in vitro* pancreatic cellular/tissue models. Daoud et al. (2011) examined the functions of human islet cells within the three various 3D extracellular matrices, namely, collagen I, collagen I with the ECM components fibronectin/collagen IV, and microfabricated scaffold with ECM-supplementation (Daoud et al., 2011). The researchers demonstrated that the 3D ECM containing the ECM components extended the life span of human islet culture. Surprisingly, the microfabricated scaffold with ECM-supplementation presented an insulin release behavior that is identical to that of the freshly isolated pancreatic islets (Daoud et al., 2011).

Kim et al. (2019) conducted a study and proposed that the pancreatic tissue-derived extracellular matrix can function as a possible candidate to mimic the native microenvironment in transplantable 3D pancreatic tissues through the observations from their research (Kim et al., 2019).

Concurrently, Klak et al. (2023) biofabricated 3D bionic scaffolds of islet cells with dECM (decellularized extracellular matrix)-based bioink using an extrusion-based 3D bioprinter, and investigated its activity on the murine models (Klak et al., 2023). The 3D bioprinted pancreatic petals were found to continue the secretion of insulin and neovascularization after transplantation, thereby dropping the plasma glucose concentration in the murine models (Klak et al., 2023). Also, Kavand et al. (2023) constructed a microstructure that can hold pancreatic islets in a specific location within the anterior chamber of the eye (ACE) and promote tissue engraftment (Kavand et al., 2023). Chen et al. (2022) reported the fabrication of 3D printed mini-capsule device containing islets that can be used for the delivery of islet cells as a treatment of T1D (Chen et al., 2022). DLP-based printing method with an exposure time of 20 s (the exposure time was confined to 20 s in order to ensure long-term survival of the islet cells) was adopted to construct the mini-capsule devices with a groove structure (the groove structure prevents the leakage of islet cells due to gravity settlement) using gelatin methacrylate (GelMA) (Chen et al., 2022). Upon subcutaneous transplantation of the encapsulated islet cells into an immunocompetent mice model, the researchers observed that the islet cells reduced the Foreign Body Reaction (FBR) from host to graft and also reduced the hyperglycemic symptoms without using immunosuppressant for long-term proving to be an effective treatment for T1D (Chen et al., 2022). Similarly, Hwang et al. (2021) designed a 3D bioprinted hybrid encapsulation system containing pancreatic islets as a treatment strategy for T1D (Hwang et al., 2021). The hybrid encapsulation system comprises an outer part polymer capsule (microporous in nature) and an inner part dECM hydrogel with pancreatic islet-like aggregates (nanoporous in nature). The 3D bioprinted hybrid encapsulation system was proven to possess enhanced therapeutic potential by directing structural maturation and functional enhancement of cells (Hwang et al., 2021).

#### 3.3.2 Management of diabetes-associated bone defects

The hyperglycemic condition and altered metabolism in T1D and T2D patients enhance the risk of fracture, impairs fracture healing and interferes with bone forming process (Jiao et al., 2015; Xu et al., 2023). Diabetic micromilieu with chronic inflammation and Diabetic Infectious Micromilieu (DIM) play a major role in the impairment of a bone regeneration process (Li et al., 2022; Xu et al., 2023). Recently, Jiang et al. (2023) 3D bioprinted the scaffolds made up of GelMA and strontium (Sr)-holding bioactive glass nanoparticles (Sr-MBGNs: mesoporous in nature) with a goal of remodeling the pathological diabetic micromilieu into immunomodulating nanocomposite with enhanced angiogenic, osteogenic and anti-inflammatory properties. The researchers observed that the Sr-MBGNs aided as a biomineralization precursor, in turn stimulated the release of calcium, silicon and strontium ions, thereby ensuring bone regeneration efficiency (Xu et al., 2023).

Interestingly, Yang et al. (2021) developed a 3D bioprinted enzyme-functionalized scaffold that performs multiple functions such as angiogenesis, anti-inflammation and osteogenesis (Yang et al., 2021). The so-called “multifunctional scaffold” was composed of alginate polymer, catalase-assisted biomineralized calcium phosphate nanosheets (CaP@CAT NSs) and glucose oxidase (GOx) enzyme. The alginate offered mechanical strength to the scaffold while GOx reduced the hyperglycemic environment by oxidizing glucose into gluconic acid and hydrogen peroxide (H_2_O_2_). The CaP@CAT NSs in scaffold was an important constituent of the scaffold for the scavenging of H_2_O_2_ molecules. This overall process induced creation of a hypoxic microenvironment, which in turn stimulated neovascularization, thereby promoting bone regeneration (Yang et al., 2021).

Osteogenesis inhibition caused by the stem cell dysfunction is another factor responsible for the impairment of a bone regeneration process in a diabetic patient (Chen et al., 2022). Researchers have observed that activation of Glycogen Synthase Kinase-3β (GSK-3β) by high glucose leads to osteogenesis inhibition by stem cell dysfunction (Zhang et al., 2016; Chen et al., 2021; Chen et al., 2022). As a strategy to promote osteogenesis by inhibiting GSK-3β, Chen et al. (2022) fabricated lithium-based mesoporous bioactive glass (for *in vitro* study) and lithium-based mesoporous bioactive glass/Poly Lactic-co-Glycolic Acid (PLGA) composite scaffold by 3D printing (for study in diabetic mice), followed by investigation of osteogenesis effect of the scaffolds (Chen et al., 2022). The researchers concluded that the scaffolds were effective enough in reversing the suppression of osteogenic differentiation by augmentation of Itga3 and activation of β-catenin/Tcf7/Ccn4 signaling pathway (Chen et al., 2022).

“Critical-sized bone defect” is a kind of bone defect that fails to heal spontaneously without surgical intervention because of the fact that its size surpasses its intrinsic healing potential (Schemitsch, 2017; Roddy et al., 2018). Sun et al. (2021) fabricated 3D bioprinted scaffolds using bioink composed of polymers gelatin, GelMA, 4-arm poly (ethylene glycol) acrylate (PEG), BMP-4 (Bone Morphogenetic Protein-4)-loaded mesoporous silica nanoparticles (MSNs), RAW264.7 macrophages (a macrophage cell line) and BMSCs (bone marrow stromal cells) (Sun et al., 2021). The researchers observed that the implantation of the scaffold in calvarial critical-size defect models of diabetic rats promoted osteogenic differentiation of BMSCs and this enhanced the bone repair in rat models (Sun et al., 2021).

### 3.4 3D drug testing models

3D *in vitro* models are the excellent therapeutic screening tools as they can mimic the exact the microenvironment of biological system. Kim et al. (2021) fabricated 3D bioprinted *in vitro* models of diseased skin containing the pathophysiological hallmarks such as the adipocyte hypertrophy, inflammatory reactions, insulin resistance and vascular dysfunction found explicitly in patients with T2D (Kim et al., 2021). The developed *in vitro* model was found to exhibit slow re-epithelization is one of the important hallmarks of diabetic skin. The research team surmised that upon stimulating an intercellular crosstalk between epidermis and dermis, the normal keratinocytes could be differentiated into diabetic epidermis by interacting with diabetic dermis (Kim et al., 2021). The researchers illustrated that this diabetic skin model can act as a perfect disease model for its use in drug development (Kim et al., 2021).

## 4 Challenges and future perspectives

The 3D printing and bioprinting technologies are expensive. This factor limits their affordability for their use in the manufacture of 3D printed medicines/bioprinted organs for public use. Most of the research related to 3D printing and bioprinting are still limited to lab scale and translational research needs to be given more importance among the researchers. Importance should be given towards understanding the impact of 3D printed and bioprinted products in biological systems by conducting pre-clinical studies and case studies wherever applicable. Secondly, though 3D printing technology can afford fabrication of pharmaceuticals with varying sizes and shapes, the production of anti-diabetic drug formulations with customized doses depending on the age and other factors of patients has not been explored till date. In the current scenario, research and development on personalization or individualization is sparse. Hence, significant importance ought to be provided to the development of patient-centric pharmaceutical/biomedical products rather than the “one-size-fits-all” medication types. Thirdly, efforts have to be improved to obtain Food and Drug Administration (FDA)-approval for the 3D printed and bioprinted products. Till date, Spritam is the only 3D printed medicine that has been approved for use by the Food and Drug Administration (FDA). Therefore, more emphasize need to be given towards 3D printed/bioprinted product development, marketing and its utility for public use. *In vitro* drug testing models, identification of solutions for the ethical issues against the use of 3D printed or bioprinted products is another area to be focused on.
